# Clinical curative effect of neoadjuvant chemotherapy combined with immunotherapy and its impact on immunological function and the expression of ER, PR, HER-2 & SATB1 in HER-2-Positive breast cancer patients

**DOI:** 10.12669/pjms.38.3.5199

**Published:** 2022

**Authors:** Zhi Chen, Mei-xiang Sang, Cui-zhi Geng, Wei Hao, Hui-qun Jia

**Affiliations:** 1 Zhi Chen, Department of Anesthesiology, The Fourth Affiliated Hospital of Hebei Medical University, Shijiazhuang, Hebei, 050017, China; 2 Mei-xiang Sang, Research Center and Tumor Research Institute, The Fourth Affiliated Hospital of Hebei Medical University, Shijiazhuang, Hebei, 050017, China; 3 Cui-zhi Geng, Breast Disease Diagnostic and Therapeutic Center, The Fourth Affiliated Hospital of Hebei Medical University, Shijiazhuang, Hebei, 050017, China; 4 Wei Hao, Baoding Third Central Hospital, Baoding, Hebei, 071051 China; 5 Hui-qun Jia, Department of Anesthesiology, The Fourth Affiliated Hospital of Hebei Medical University, Shijiazhuang, Hebei, 050017, China

**Keywords:** Breast cancer, HER-2 positive, Immunotherapy, Neoadjuvant chemotherapy

## Abstract

**Objective:**

To evaluate the clinical curative effect of neoadjuvant chemotherapy combined with immunotherapy and its impact on immunological function and the expression of ER, PR, HER-2 and SATB1 in HER-2-positive breast cancer patients.

**Methods:**

The subjects of study were 80 patients with HER-2-positive breast cancer. Enrolled patients were randomly divided into two groups, with 40 cases in each group at The Fourth Affiliated Hospital of Hebei Medical University from March 2018 from March 2021. Patients in the control group were provided with neoadjuvant chemotherapy using TAC regimen merely; while those in the study group received oral administration of Apatinib Mesylate (500mg/d; three weeks a cycle) on the basis of the TAC regimen. Further comparative analysis was performed focusing on the therapeutic effect and adverse drug reaction rate of the two groups; levels of CD3^+^, CD4^+^, CD8^+^ and CD4^+^/CD8^+^ of T lymphocyte subsets in the two groups before and after treatment; as well as the expressions of ER, PR, HER-2 and SATB1 in the two groups before and after treatment.

**Results:**

The total response rate was 77.5% and 55% in the study group and the control group, respectively, with an obviously better outcome in the former group than that in the latter group (p=0.03). Meanwhile, the incidence of adverse reactions was 40% in the study group and 45% in the control group, without statistical difference (p=0.65). There were statistically significant differences that the levels of CD3^+^, CD4^+^, and CD4^+^/CD8^+^ in the study group were significantly higher when compared with those in the control group after treatment (CD3^+^, p=0.00; CD4^+^, p=0.02; CD4^+^/CD8^+^, p=0.00); while no evident change was observed in the level of CD8^+^ (p=0.88). After treatment, the positive expression rates of ER, HER-2 and SATB1 were remarkably lower in the study group than those in the control group, showing statistically significant differences (ER, HER-2, p=0.03; SATB1, p=0.02). However, there was no statistically significant difference in the positive expression rate of PR between the study group and the control group (P=0.80).

**Conclusions:**

Neoadjuvant chemotherapy combined with immunotherapy has significant effect on the treatment of HER-2-positive breast cancer patients. It can result in the significant enhancement of T lymphocyte function, obvious improvement in the negative converse rates of ER, HER-2 and SATB1, and no evident increase in the adverse drug reactions. The proposed therapeutic approach is safe, effective, and have certain clinical value.

## INTRODUCTION

Breast cancer is the most frequently diagnosed female malignancy, and the second most common cause of cancer related death in women.^1^ It shows an increased incidence in younger population in recent decades, and approximately 1/8 of the people have breast cancer in developed countries.^2^ Breast cancer is characterized by a hidden onset and no obvious symptoms at the early stage. Most patients have developed to the late stage at diagnosis, and missed the optimal opportunity for treatment. According to a prior research by Goto et al.^3^, about 15% of patients with breast cancer have metastasis at the time of diagnosis. Breast cancer is developed owing to the involvement of multiple factors, therefore, its treatment requires the combination of multiple therapeutic approaches.^4^

At present, the combined use of surgery and chemotherapy is a common choice for the treatment of breast cancer in the clinical setting. Furthermore, human epidermal growth factor receptor-2 (HER2) is overexpressed in around 20% of breast cancer. Patients with HER2-positive breast cancer may experience a rather poor prognostic outcome. With the emergence of HER2-targeted monoclonal antibodies (Trastuzumab and Pertuzumab), there is a great change in the clinical outcome of HER2-positive breast cancer. However, it remains to be a major therapeutic challenge in terms of the resistance to HER2 therapy.^5^ Meanwhile, neoadjuvant chemotherapy is a medical regimen used prior to surgical resection of tumors.

Currently, neoadjuvant chemotherapy is generally adopted for the treatment of patients with locally advanced breast cancer and breast cancer patients who need to benefit from tumor volume reduction before surgery. At present, there is sufficient evidence supporting that patients may experience better therapeutic outcome when there is a complete pathological response to neoadjuvant chemotherapy.^6^ Importantly, as recommended by Gianni et al.^7^, for female patients with HER2-positive breast cancer, neoadjuvant chemotherapy associated with trastuzumab as adjuvant therapy should be considered to improve the event-free survival rate, survival rate as well as clinical and pathological tumor response.

In our study, neoadjuvant chemotherapy combined with immunotherapy was applied for treatment with the achievement of certain therapeutic benefit, which can improve the clinical curative effect, enhance the immune function, and reduce the positive expression rate of ER, HER-2 and SATB1 in patients with HER-2-positive breast cancer. It is reported as follows:

## METHODS

A total of 80 cases of HER-2positive breast cancer patients in our hospital were selected and randomly divided into two groups, with 40 cases in each group at The Fourth Affiliated Hospital of Hebei Medical University from March 2018 from March 2021. The age of the study group was 43~68 years old, with an average of (55.26±9.50) years old, and that of the control group was 41~70 years, with an average of (56.68±9.93) years old. There was no significant difference in general data between the two groups, showing a comparability between the two groups (Table-I).

**Table-I:** Comparative analysis of general data between the study group and the control group (*x*¯±S, n=40 in each group).

Indexes	Study group	Control group	t/χ^2^	p
Age (years old)	55.26±9.50	56.68±9.93	0.65	0.51
***Clinical stage***				
Stage II (%)	25	27	0.22	0.64
Stage III (%)	15	13		
Tumor diameter (cm)	2.30±0.74	2.27±0.68	0.19	0.85
***Tumor location***				
Left (%)	21	23	0.20	0.65
Right (%)	19	17		
***Pathological type***				
Ductal carcinoma	32	34	0.35	0.56
Lobular carcinoma	4	4	0.00	1.00
Others	4	2	0.72	0.40

p>0.05.

### Ethical Approval

The study was approved by the Institutional Ethics Committee of The Fourth Affiliated Hospital of Hebei Medical University on March 25, 2018(No.HDFY-LL-2018-039), and written informed consent was obtained from all participants.

### Inclusion criteria:

1. Patients with breast cancer confirmed by histopathological biopsy^8^, and with positive HER-2 by immunohistochemistry;
2. Patients who aged ≤70 years old;
3. Patients who were found to have clear lesions by imaging examination, with accurately measurable size of the lesions;
4. Patients with complete clinical data who received treatment for the first time;
5. Patients with KPS score ≥70 points, and the predicted survival period ≥12 months;
6. Patients with TNM stage II~III, without metastasis and with indications of surgery;
7. Patients with good treatment compliance that the patients and their families were willing and able to cooperate to complete the study;
8. Patients without contraindications to the drugs used in this study;
9. Patients who knew the contents of this study in advance, voluntarily participated in the study and signed informed consent.

### Exclusion criteria:

1. Patients with severe cardiopulmonary dysfunction;
2. Patients with other malignant tumors at the same time;
3. Patients with abnormal mental or cognitive function who could not cooperate to complete the study;
4. Patients who have occurred or complicated with serious complications and could not tolerate the operation;
5. Patients who take relevant drugs (immunosuppressants, hormones, etc.) recently that might affect the study.

### Therapeutic Methods

Blood cell analysis, liver function and renal function examinations were performed in both groups before treatment. According to the results, the obviously abnormal indexes were corrected, and the patients with malnutrition were provided with nutritional support. Water, electrolyte and acid-base balance were maintained during treatment. Hydration was adopted the day before chemotherapy.

Patients in both groups received neoadjuvant chemotherapy by using TAC regimen^9^, with the specific regimen described as follows: Docetaxel (75 mg/m^2^) through intravenous drip, D1; cyclophosphamide (500 mg/m^2^) and epirubicin (70 mg/m^2^), D2~D21; with 21 days as a cycle. Patients in the study group received oral administration of Apatinib Mesylate (500 mg/d; three weeks a cycle, continuous use) on the basis of the TAC regimen.

### Observational indexes:

### Curative effect evaluation

Curative effect of all patients was evaluated after every two treatment cycles. The tumor was evaluated according to the Response Evaluation Criteria In Solid Tumors 1.0 (RECIST1.0) standards^10^: complete remission (CR): complete disappearance of the lesion; partial remission (PR): decrease of the tumor volume of >75% after treatment; stable disease (SD): decrease of the tumor volume of between 50~75% after treatment; and Progressed disease (PD): no decrease of the tumor volume or even increase and the appearance of new lesion after treatment. Overall response rate = Number of cases with (CR+PR)/Total cases×100%.

### Adverse drug reaction assessment

The adverse drug reactions (bone marrow suppression, gastrointestinal reaction, peripheral neuritis, liver function injury, etc.) were recorded after the treatment cycle of the two groups. Immune status analysis: Blood samples were taken from patients in a fasting condition before and after treatment to detect the levels of CD3^+^, CD4^+^, CD8^+^, and CD4^+^/CD8^+^ T lymphocyte subsets. Further comparative analysis was carried out focusing on the differences between the two groups before and after treatment. Comparative analysis of ER, PR, HER-2 and SATB1 expressions: All patients underwent tissue biopsy using hollow needle before treatment and after chemotherapy to collect the specimens for detection. The expressions of ER, PR, HER-2 and SATB1 were measured before and after treatment by immunohistochemistry (IHC). The positive expressions of ER, PR, HER-2 and SATB1 were observed under five high-power microscopic view. The number of positive cells and staining intensity were judged by semi-quantitative integral method. The final results were determined by two senior pathologists.

### Statistical Analysis

All data in this study were analyzed by SPSS 20.0 software. Measurement data were expressed in (*x¯*±S). Inter-group data comparison utilized independent sample t-test, and paired t-test was used for intra-group data analysis; while the comparison of rate used χ^2^ test. P<0.05 meant that the difference was statistically significant.

## RESULTS

The comparative analysis of treatment effects in patients between the two groups is shown in Table-II. After treatment, the overall response rate of the study group was 77.5% and that of the control group was 55%. It was significantly better in the study group when compared with the control group, with statistically significant difference (p=0.03).

**Table-II:** Comparative analysis of therapeutic effect between the two groups (*x*¯±S, n=40 in each group).

Groups	CR	PR	SD	PD	Overall response rate
Study group	9	22	4	5	31 (77.5%)
Control group	5	17	13	5	22 (55%)
χ^2^					4.53
p					0.03

p<0.05.

According to the comparative analysis results of adverse drug reactions between the two groups after treatment (Table-III), the rate of adverse reactions was 40% and 45% in the study group and the control group, respectively. No statistically significant difference was observed in rate of adverse reactions between the study group and the control group (p=0.65).

**Table-III:** Comparative analysis of adverse drug reactions between the two groups after treatment (*x*¯±S, n=40 in each group).

Groups	Bone marrow suppression	Gastrointestinal reaction	Peripheral neuritis	Liver function injury	Incidence rate
Study group	3	5	2	6	16 (40%)
Control group	4	4	3	7	18 (45%)
χ^2^					0.20
p					0.65

p>0.05

Before treatment, there was no significant difference in the levels of CD3^+^, CD4^+^, CD8^+^, and CD4^+^/CD8^+^ in both groups (p>0.05). While after treatment, the levels of CD3^+^, CD4^+^, and CD4^+^/CD8^+^ were highly increased in the study group than those in the control group, with statistically significant differences (CD3^+^, p=0.00; CD4^+^, p=0.02; CD4^+^/CD8^+^, p=0.00). However, no obvious change was found in the level of CD8+ (p=0.88), as shown in Table-IV. There was no significant difference in the positive expression rates of ER, PR, HER-2 and SATB1 between the two groups before treatment (P>0.05). Furthermore, in terms of the re-detection results after treatment, the positive expression rates of ER, HER-2 and SATB1 in the study group were significantly lower than those in the control group, and the differences were statistically significant (ER, HER-2, p=0.03; SATB1, p=0.02); While no statistically significant difference was noticed in PR positive expression rate between the study group and the control group (p=0.80). The results are shown in Table-V.

**Table-IV:** Comparative analysis of T lymphocyte subsets between the two groups before and after treatment (*x*¯±S, n=40 in each group).

Indexes		Study groupΔ	Control groupΔ	t	p
CD3+ (%)	Before treatment*	41.35±5.72	41.66±6.01	0.24	0.81
	After treatmentΔ	49.47±6.83	45.19±7.01	2.77	0.00
	t	5.76	2.42		
	p	0.00	0.02		
CD4+ (%)	Before treatment*	26.21±4.46	26.32±4.82	0.11	0.92
	After treatmentΔ	37.51±5.25	34.62±5.73	2.35	0.02
	t	10.37	7.01		
	p	0.00	0.00	
CD8+ (%)	Before treatment*	23.06±4.75	23.64±3.78	0.60	0.55
	After treatment*	23.88±4.67	23.73±4.23	0.15	0.88
	t	0.78	0.10		
	p	0.44	0.92		
CD4+/CD8+	Before treatment*	1.46±0.31	1.47±0.23	0.15	0.88
	After treatmentΔ	1.97±0.28	1.65±0.41	4.46	0.00
	t	7.72	2.27		
	p	0.00	0.03		

* p>0.05, Δp<0.05.

**Table-V:** Comparative analysis of the expression of ER, PR, HER-2 and SATB1 between the two groups before and after treatment (*x*¯±S, n=40 in each group).

Indexes		Study group	Control group	t	p
ER positive rate (%)	Before treatment	19 (47.5%)	17 (42.5%)	0.20	0.65
	After treatment	8 (20%)	16 (40%)	4.71	0.03
PR positive rate (%)	Before treatment	16 (40%)	18 (45%)	0.20	0.65
	After treatment	14 (35%)	15 (37.5%)	0.06	0.80
HER-2 positive rate (%)	Before treatment	40 (100%)	40 (100%)	0.00	1.00
	After treatment*	10 (25%)	19 (47.5%)	4.38	0.03
SATB1 positive rate (%)	Before treatment	28 (70%)	27 (67.5%)	0.06	0.81
	After treatment*	12 (30%)	22 (55%)	5.12	0.02

* p<0.05.

## DISCUSSION

Globally, breast cancer is still such a malignancy with the highest incidence rate in females, accounting for 30% of all newly-onset cases in women. Worldwide, breast cancer is the leading cause of death among women, and its incidence rate is rising in low-income countries.^11^ In terms of the pathogenesis of breast cancer, it can be attributed primarily to the proliferation of breast epithelial cells caused by multiple carcinogenic factors. The common therapies used for breast cancer include surgery, chemotherapy, radiotherapy, etc. With the development of medical technology, surgical treatment is on longer the single choice for breast cancer. It becomes a mainstream by using comprehensive therapy based on the combination of surgery and multiple adjuvant treatments. Traditionally, the therapeutic regimens of breast cancer clinically are developed mainly based on tumor size, pathological type, lymph node metastasis, etc. Along with the accumulation of genomic analysis and clinical data, it provides a new direction to formulate different treatment regimens according to the different expression of HER-2 in breast cancer patients.^12^

Around 30% are HER-2-positive among breast cancer patients.^13^ HER-2 gene is a proto-oncogene, which is inactive under normal conditions. It is mainly involved in the growth and reproduction of body tissues and cells. Carcinogenic factors can lead to the overexpression of HER-2, which can further promote tumor cell proliferation and malignant transformation, increase tumor cell invasiveness, and result in higher risk of recurrence and metastasis.^14^ By targeting HER-2, a first-line treatment regimen represented by Trastuzumab and Pertuzumab was established in 2012.^15^ Specifically, Trastuzumab is a novel human HER-2-targeted antibody drug conjugate (ADC) with topoisomerase I inhibitor payloads.^16^ However, this treatment scheme possesses certain defects, which is mainly manifested as the presence of resistance to HER-2 therapy, resulting in obviously reduced therapeutic effect.

Neoadjuvant chemotherapy is the standard treatment for advanced breast cancer, which is a chemotherapy regimen adopted before surgical resection. The combined application of docetaxel, cyclophosphamide and epirubicin can significantly improve the effect of chemotherapy, increase the CR rate of tumor and enhance the survival of relevant patients. In addition to a role in killing tumor cells, neoadjuvant chemotherapy can also promote the transformation of tumor cells into normal breast cells, so as to gradually restore the expression of ER, HER-2, SATB1 and other indexes to the normal range^17^ However, it should be noted that the immune dysfunction caused by chemotherapy will also result in immunosuppression of patients and reduce the therapeutic effect. In view of previous investigation, Pop et al.^18^ believed that neoadjuvant chemotherapy combined with immunotherapy could achieve a relatively higher pathological complete remission rate. Meanwhile, Hassett et al.^19^ reported that combined application of immunotherapy from the beginning of neoadjuvant chemotherapy would reduce the therapeutic cost, and reduce the dose and toxicity of chemotherapeutics. Besides, it could significantly improve the event-free survival period and overall survival (OS).^20^

Apatinib is a novel small molecule, selective vascular endothelial growth factor receptor-2 (VEGFR-2) tyrosine kinase inhibitor. It can be used for the treatment of advanced gastric adenocarcinoma, non-small cell lung cancer (NSCLC), breast cancer, gynecologic cancer, hepatocellular carcinoma (HCC), thyroid cancer and sarcoma.^21^ Apatinib could significantly prolong the median progression-free survival (PFS) and OS of patients^22^, especially for tumor patients with high microsatellite instability, exhibiting incomparable advantages over chemotherapeutics.^23^ For instance, Maroufi et al.^24^ reported in their research that Apatinib can can inhibit the proliferation, migration and invasion of breast cancer cell line MDA-MB-231 by inducing apoptosis, cell cycle arrest, regulating NF-κB and MAPK signaling pathways, and thus playing a role in the treatment of breast cancer. Its combined use with chemotherapy can significantly improve the immune state of the body, yet without obvious increase in adverse reactions.^25^ In addition, Geng et al.^26^ considered that the adverse reactions of antiangiogenic drugs might indicate the effectiveness of these drugs.

In our study, the overall response rate of neoadjuvant chemotherapy by using TAC regimen combined with Apatinib Mesylate was 77.5% and that of the control group was 55% (p=0.03). Meanwhile, the incidence of adverse reactions was 40% in the study group and 45% in the control group, without statistical difference (p=0.65). There were statistically significant differences that the levels of CD3^+^, CD4^+^, and CD4^+^/CD8^+^ in the study group were significantly higher when compared with those in the control group after treatment (CD3^+^, p=0.00; CD4^+^, p=0.02; CD4^+^/CD8^+^, p=0.00). After treatment, the positive expression rates of ER, HER-2 and SATB1 were remarkably lower in the study group than those in the control group, showing statistically significant differences (ER, HER-2, p=0.03; SATB1, p=0.02).

### Limitations of the study

It includes smal sample size, relatively shorter period of follow-up, and no inclusion of surgery-related indicators in this study. With the expansion of sample size, our Research Group will continue to improve the surgery-related indicators, expand the follow-up content and prolong the duration of follow-up. It is expected to make a more objective evaluation of the impact of the therapeutic regimen on surgery and its long-term therapeutic effect, etc.

## CONCLUSIONS

To sum up, neoadjuvant chemotherapy combined with immunotherapy has significant effect on the treatment of HER-2-positive breast cancer patients. It can result in the significant enhancement of T lymphocyte function, obvious improvement in the negative converse rates of ER, HER-2 and SATB1, and no evident increase in the adverse drug reactions. The proposed therapeutic approach is safe, effective, and have certain clinical value.

### Authors’ Contributions:

**ZC &** **HQJ:** Carried out the studies, participated in collecting the data, drafted the manuscript and were responsible and accountable for the accuracy and integrity of the work.

**MXS & CZG:** Performed the statistical analysis and participated in the study design.

**WH: S**significantly revised this manuscript and participated in the study.
